# Watch Out for Your Neighbor: Climbing onto Shrubs Is Related to Risk of Cannibalism in the Scorpion *Buthus* cf. *occitanus*

**DOI:** 10.1371/journal.pone.0161747

**Published:** 2016-09-21

**Authors:** Francisco Sánchez-Piñero, Fernando Urbano-Tenorio

**Affiliations:** Dpto. de Zoología, Facultad de Ciencias, Universidad de Granada, 18071, Granada, Spain; Ben-Gurion University of the Negev, ISRAEL

## Abstract

The distribution and behavior of foraging animals usually imply a balance between resource availability and predation risk. In some predators such as scorpions, cannibalism constitutes an important mortality factor determining their ecology and behavior. Climbing on vegetation by scorpions has been related both to prey availability and to predation (cannibalism) risk. We tested different hypotheses proposed to explain climbing on vegetation by scorpions. We analyzed shrub climbing in *Buthus* cf. *occitanus* with regard to the following: a) better suitability of prey size for scorpions foraging on shrubs than on the ground, b) selection of shrub species with higher prey load, c) seasonal variations in prey availability on shrubs, and d) whether or not cannibalism risk on the ground increases the frequency of shrub climbing. Prey availability on shrubs was compared by estimating prey abundance in sticky traps placed in shrubs. A prey sample from shrubs was measured to compare prey size. Scorpions were sampled in six plots (50 m x 10 m) to estimate the proportion of individuals climbing on shrubs. Size difference and distance between individuals and their closest scorpion neighbor were measured to assess cannibalism risk. The results showed that mean prey size was two-fold larger on the ground. Selection of particular shrub species was not related to prey availability. Seasonal variations in the number of scorpions on shrubs were related to the number of active scorpions, but not with fluctuations in prey availability. Size differences between a scorpion and its nearest neighbor were positively related with a higher probability for a scorpion to climb onto a shrub when at a disadvantage, but distance was not significantly related. These results do not support hypotheses explaining shrub climbing based on resource availability. By contrast, our results provide evidence that shrub climbing is related to cannibalism risk.

## Introduction

Foraging animals balance resource finding and acquisition in response to food availability and predation risk [1], [2], [3]. As a consequence, predators affect prey populations not only directly by killing and consumption, but also indirectly through nonlethal effects on prey behavior and spatial distribution [3], [4]. Thus, predation threat becomes an important factor determining where animals can feed safely, affecting trophic interactions as well as population and community structure [4], [5].

In predators with size-structured populations, cannibalism is usually an important interaction shaping the distribution, behavior, and population structure of these species [6], [7], [8]. Cannibalism is an asymmetric interaction usually involving a larger-sized predator feeding on smaller conspecifics [6]. Therefore, cannibals are relatively invulnerable to injury and death during a predatory attack. Cannibalism has been hypothesized to evolve when selection pressures favoring it exceed its costs [6], [9]. Benefits include energy gain and removal of a competitor, while costs are mainly risks of injury or predation, infection from parasites and diseases, and loss of inclusive fitness. The consequences of cannibalism are polyphenism and selection for diverse behavioral traits [6], [9]. Thus, responses to avoid or minimize cannibalism such as the use of (micro-)habitats and activity periods that exhibit low risk of cannibalism may have evolved in cannibalistic species [10], [11]. However, few studies have focused on whether cannibalism affects foraging behavior and microhabitat use [6], [9].

Cannibalism, an important interaction in scorpions, is together with intraguild predation (by other scorpion species) one of the primary sources of mortality, while vertebrate and other large invertebrate predators are probably secondary [12]. Cannibalism has been shown to be the main cause of mortality among smaller and/or immature animals [6], [12], [13], [14], with conspecifics representing as much as 5.6–9.3% of prey in the diet and 30% of the ingested biomass [12], [14], [15], [16]. Diverse behavioral responses have been related to the reduction of predation by conspecifics, including divergence in seasonal and diel activity periods, (micro-)habitat selection, and climbing on vegetation [17], [18], [19], [20].

Shrub climbing by small scorpion species and younger age classes of larger scorpion species has been hypothesized both as a foraging strategy to maximize prey capture and as a response to reduce predation risk, especially cannibalism by larger scorpions [20], [21], [22], [23]. Brown and O’Connell [23] and McReynolds [20] studied climbing on vegetation by the scorpion *Centruroides vittatus* (Say, 1821). In no case was conclusive evidence found to support the use of vegetation as a means to increase prey capture, hence suggesting that climbing on plants may constitute a predator-avoidance behavior. However, Sánchez-Piñero et al. [24] hypothesized that foraging on shrubs by *Buthus* cf. *occitanus* could be related to the availability of smaller, more suitable prey for small scorpions on shrubs, suggesting also that shrub selection by this scorpion species could depend on prey abundance on the different shrubs, as proposed by McReynolds [20] for *C*. *vittatus*. However, none of these works have analyzed shrub climbing in relation to predation risk.

In the present study, we analyze climbing on shrubs by the scorpion *Buthus* cf. *occitanus* (hereafter *B*. *occitanus*) in relation to prey availability and predation risk (cannibalism). We provide for the first time empirical evidence supporting the relationship between the foraging behavior of the scorpion and the risk of cannibalism. This scorpion species is known to climb and forage on shrubs, with as many as 40% of the individuals, mainly juveniles, found up to 80 cm high on the canopy of shrubs [22], [24], [25]. At the Guadix-Baza Basin, Sánchez-Piñero et al. [20] found ca. 40% of scorpions on shrubs, showing that climbing on vegetation was inversely related to scorpion size. For the smallest scorpions (10–20 mm length), 46% were observed on shrubs while 34% of the individuals measuring > 20 mm to 50 mm length and none of the individuals > 50 mm length were found on shrubs. In agreement with Polis [13], [14], reduction in the risk of cannibalism by adults is likely related to juvenile foraging strategies, such as climbing on vegetation, while predation risk from predators relying on visual cues does not appear to be a key factor affecting the foraging of juvenile *B*. *occitanus* [22]. Specifically, we tested the following hypotheses: 1) small scorpions find and capture small, more suitable, prey on shrubs than on the ground [23], [24]; 2) selection of particular shrubs is related to prey availability [20], [24]; 3) seasonal variations in shrub climbing are correlated with fluctuations in prey availability [20], [23]; 4) shrub climbing is related to cannibalism risk [15], [21], [22].

### Biology of *B*. *occitanus*

The *Buthus occitanus* (Amoreux 1789) species complex is a group of closely related species formerly considered as a single species widely distributed around the Mediterranean region and North Africa [26], [27], [28]. In the Iberian Peninsula several lineages and species of this group have been described recently [28], [29], [30]. In the analysis by Sousa et al. [30] scorpions from the study area (Sc 96) appear as a distinct lineage closely related to a North African species [*B*. *tunetanus* (Herbst, 1800)], a result requiring confirmation.

*Buthus occitanus* (*sensu lato*) comprise large-sized species with foraging individuals ranging from 13 mm for the smallest juveniles to 60–70 mm (and up to 85 mm) of adults. They are generalist predators, feeding on insects (adults and larvae) and arachnids (spiders, solifuges and conspecifics) [24], [25], [31]. Foraging by *B*. *occitanus* is highly flexible to exploit available resources [31]. They can capture prey on the ground surface, on shrubs [24], and below ground where they detect prey close to the surface and dig to capture them (F.S.P. and F.U.T., pers. obs.). The diet for scorpions at the study site includes Araneae, *B*. *occitanus* conspecifics, Hemiptera, adult and larval Coleoptera (except for large adult tenebrionids), Formicidae, Embioptera, adult and larval Lepidoptera, Lepismatidae, larval Raphidioptera, Scolopendromorpha, and Solifugae [24], [S1 Table].

As in most scorpion species, *B*. *occitanus* is a nocturnal sit-and-wait forager, remaining in one place until a prey is close enough to capture it [21], [22], [32]. Nonetheless, our observations indicate that scorpions can move to a different place if they are unsuccessful capturing prey. Adult females usually forage near their burrows or at the entrance (doorkeeping behavior). During the reproductive period, mainly at the beginning of the activity season, males are mobile and vagrant, apparently searching for mates. As in other scorpions, only a proportion of individuals in a population are active on a given night [15], [21], [32].

## Methods

### Study area

The study was conducted at Barranco del Espartal (37.53173°N, 2.69623°W to 37.53603°N, 2.69949°W, 750 m altitude), an occasional water course located in the arid Guadix-Baza Basin (Granada, southeastern Spain). The site is a privately owned area, and permission from the owners and Consejería de Agricultura, Pesca y Medio Ambiente, Junta de Andalucía, allowed us to conduct this research. Potential evapotranspiration in the area exceeds, by a factor of three, the amount of annual rainfall (250–300 mm). The climate is Mediterranean continental, with strong temperature fluctuations (ranging from 40 to -14°C), and marked seasonality. The substrate is slightly calcareous, composed of silt mixed with gypsum sediment.

The vegetation is an arid open shrub-steppe (58% bare soil, 41% shrub cover) dominated by chamaephyte shrubs (*Artemisia herba-alba* Asso, *A*. *barrelieri* Bess and *Salsola vermiculata* L.), tussock grasses (*Stipa tenacissima* L. and *Lygeum spartum* L.) and phanerophyte bushes (*Retama sphaerocarpa* L.). Shrubs in the study area are randomly distributed, and individual plants usually average 0.5 m apart [33]. A more detailed description of the study site can be found elsewhere [34].

### Sampling design

To estimate the proportion of scorpions on shrubs and on the ground, and the size structure of the scorpion population, we sampled scorpions by exhaustive search using a black light during a minimum of 8–10 s/m^2^ to avoid detection biases of small individuals or individuals in or under shrubs. Censuses were conducted in a total of six randomly selected 50 m x 10 m plots in plain sites around the dry riverbed within the study area. Plots were placed (by means of 0.5 m tall stakes driven into the soil) and sampled from mid-June to mid-October, the period of highest scorpion activity in the study area. Each year from 2012 to 2014, the six plots were established in the same areas, although their exact locations differed among years. Each night, after sunset and up to 3:00 h a.m., we sampled two or three plots, depending on the number of active scorpions. Plots were sampled biweekly in 2012 (except in September, when we were able to sample only once at the beginning of the month) and once a month in 2013 and 2014, due to logistic limitations.

We measured the length of each scorpion (both on the ground and on shrubs) directly in the field. Scorpions were restrained in a thick, transparent plastic bag and their tails were straightened using forceps. Body length was measured from the chelicerae to the telson, maintaining the telson folded on the metasoma, as most scorpions adopted this position. The total length of each individual was measured to the nearest mm with a small metal ruler. In addition, we recorded whether the scorpion was on the ground or on a shrub, specifying the shrub species. After measurement, each individual was released where it was found.

In 2012 and 2014 we selected individuals within and around the plots to assess whether their climbing on a shrub was related to size difference and distance between a focal scorpion and its nearest neighbor. To select individuals for the analysis, we exhaustively searched for scorpions within 2-m-wide transects across the 50 m x 10 m plots walking in zigzag to cover the area within the plots and around them. During the search, the first individual detected different from previously measured scorpions included either as “focal” or nearest neighbor was chosen. Selected individuals were measured and their position marked with a flag. Then, we looked for the nearest neighbor scorpion, and once it was found, we recorded the distance between the two scorpions. During the searchings we did not observe any changes in the behavior of scorpions due to the UV detection method. The act of finding the first scorpion did not disturb the other scorpions nearby (although we observed that large doorkeeping females sometimes retreated inside their burrows when they detected the vibrations in the soil as we approached within 1–2 m; see Discussion). Because scorpions are preyed upon by larger individuals [19], we considered that only those individuals of equal or larger size would constitute a threat. Thus, risk of cannibalism was assessed according to the size difference between a scorpion and its nearest neighbor when this individual was of equal or larger size (NELS). Scorpions whose nearest neighbors were of smaller size were excluded from the analysis, since the roles of potential cannibal and prey would be reversed. There was also a potential bias that not climbing at the largest size differences (i.e. large 50–70 mm scorpions when neighbors were small 13–20 mm scorpion) was related to physical constraints of large scorpions against climbing onto shrubs and not against cannibalism or predation risk, as hypothesized for spiders [35], [36]. In addition, we considered that the distance between scorpions could also be important, since some scorpions have specialized vibration-sensitive mechanoreceptors on tarsal leg segments which allow the detection of soil vibrations over distances of a few decimeters, from which they are able to determine the size, direction of movement of prey or potential threats, and the distance from it [37], [38], [39]. Consequently, we might expect an effect of distance between neighboring scorpions, since a larger and closer neighbor could be more easily detected and would represent a higher threat.

#### Prey availability

To assess prey abundance in the different shrub species used by *B*. *occitanus*, we used 10 cm x 10 cm sticky traps covered with glue on both sides. We employed this method because it was easier than other techniques such as shrub beating, and allowed arthropods to be collected throughout the entire night. Traps were placed at sunset and retrieved at dawn, collecting arthropods exclusively overnight in order to provide estimates of prey availability during the period of scorpion activity. Prey sampling was conducted in the plots where scorpion surveys were already performed on previous nights, or in nearby areas in plots where scorpion were not yet surveyed, in order to avoid disturbing the study plots.

Sticky traps on shrubs were placed once a month from July to October in 2012, 2013, and 2014 but, in August and September 2012, sampling was conducted twice a month. In each plot, we placed one trap in one arbitrarily selected shrub of nine or ten randomly assigned species. Therefore, one trap was placed on five individual shrubs of the eleven shrub species used by *B*. *occitanus* [24]. Thus, every two weeks or month a total of 55 traps were placed on the shrubs.

To analyze whether shrub climbing by scorpions was related to prey availability, we considered only the taxa included in the diet of the scorpion at the study site [S1 Table] for a better adjustment of prey availability with the scorpion diet [40], [41]. Insects captured by the scorpion due to attraction to black lights were not considered.

To compare the proportion of prey captured on the ground and on shrubs by scorpions, we considered only prey found being eaten by scorpions at the time of sampling which we were able to identify as either a ground or a shrub dweller, since our observations indicate that some scorpions capture prey on the ground but climb onto a shrub to feed on it. Prey classification as shrub or ground dwellers was based on published information on the arthropod fauna at the study area (see [42] and references therein), and on the information provided by specialists on spiders and hemipterans. Insects attracted to our lights that were captured by scorpions were not included.

To assess differences in prey size in shrubs and on the ground, we measured the body length of 280 prey (124 shrub, 156 ground) from sticky traps placed in shrubs and on the ground from July to October 2014, and 173 prey (83 shrub, 90 ground) collected directly in the field by means of soil sampling and shrub beating in August and September 2014. We measured prey from the sticky traps placed in shrubs to estimate prey abundance, as described above. Sticky traps on the ground were 5 cm x 20 cm plastic card covered with adhesive on the upper side. We used traps of different sizes on the ground and in vegetation because arthropods were collected almost exclusively on the periphery of the traps placed on the ground. Sticky traps both in shrubs and on the ground were placed at sunset and retrieved at down in order to collect prey available during the period of scorpion activity. Beating was used on 30 arbitrarily assigned shrubs each month, collecting the arthropods in a 20-cm-diameter funnel placed below the shrub and connected to a plastic bag, a method recommended to reduce the escape of flying insects from the samples [43]. A total of 30 soil samples were collected each month arbitrarily both on bare ground and under shrubs, since scorpions were found in both microhabitats. Samples were collected by taking 1000 cm^3^ of litter and soil in a 20 cm x 10 cm area x 5 cm depth in litter/soft soil sites or 20 cm x 20 cm area x 2.5 cm depth in harder soil sites, because scorpions are able to dig in the litter and soft soil to capture belowground dwelling larvae. Both shrub beating and soil sampling were carried out at dusk and during the first two hours of the night. Body length was measured to 0.5 mm precision, since the length of prey captured on sticky traps could not be measured with a higher accuracy.

To analyze whether scorpions used the shrub species randomly or selected for specific types of shrubs, we estimated shrub availability by counting the number of shrubs of each type in five randomly assigned 10 m x 1 m areas within each of the six plots surveyed. For the random assignment of these 10 m x 1 m areas, we divided the 50 m x 10 m plots in five 10 m x 10 m quadrats. Then, we randomly chose one of the ten 10 m x 1 m transects previously established inside each of the five 10 m x 10 m quadrats.

### Statistical analysis

Frequency distributions were compared by means of χ^2^ tests. This test was used specifically to compare the proportions of a) *B*. *occitanus* found in shrubs/on the ground among years and months, b) prey captured vs. number of scorpions in shrubs/on the ground, and c) shrub species used by scorpions among years and months.

Differences in prey availability and prey size in shrubs vs. on the ground were analyzed using nonparametric statistics, due to differences in variance and lack of fit of residuals from ANOVA and GLM models to a normal distribution. Because data were strongly heteroscedastic, differences in prey size between ground and shrubs were analyzed by the nonparametric Welch test on ranks [44]. Prey availability among shrub species was also compared by means of the Welch test on ranks, using the Ryan-Einot-Gabriel-Welsch (REGWQ) test for multiple comparisons of mean prey abundance between pairs of shrub species, as recommended by Cribbie et al. [44]. The analysis was made using the SPSS Statistics 22 package.

The relationship between phenological variations in the number of scorpions found in shrubs and prey availability was analyzed by means of a GLM test with a normal distribution and identity link including the total number of scorpions as the covariate, to correct for the changes in the number of active scorpions during the study. Variables were logarithmically transformed to avoid overdispersion. For this analysis, we used the JMP 8 statistical package.

To analyze whether scorpions selected different species of shrubs, we quantified selectivity using the *W*_*i*_ Savage’s index. This index is the ratio between the proportion of resources used and the proportion of available resources in the environment, with index values > 1 indicating preference and index values < 1 avoidance [43]. Significance of selection was evaluated by the χ^2^ test [45], [46], with *a posteriori* correction of significance by the sequential Bonferroni procedure [47]. To determine whether selection of the different shrub species (response variable) was related to prey availability (predictor variable), we used a linear regression. *W*_*i*_ values for 2012 and 2014 were logarithmically transformed to meet the assumption of normal distribution of the residuals.

Spearmann rank correlation was used to analyze the relationship between monthly variations in prey availability and shrub selectivity. This non-parametric test was used because residuals from linear regression analysis were non-normally distributed.

To test whether the presence of scorpions on shrubs or on the ground (binary categorical dependent variable) was related to the presence of a potential cannibal, we applied a multiple logistic regression including two continuous independent variables [48]: a) size difference between a scorpion and its NELS, and b) distance between the two scorpions. The analysis was made using the JMP 8.1 package (StatSoft 2005). Although some authors have reported that moon illumination affects *B*. *occitanus* activity [22], [49], moonlight (measured as the percentage of the moon illuminated, taken from the free access application of the US Naval Observatory “*Fraction of the Moon Illuminated*”) was not included in the analysis since data showed that there was no relationship between moonlight and the probability to find a scorpion on a shrub (2012: Wald χ^2^ = 0.257, p = 0.612, d.f. = 1; 2014: Wald χ^2^ = 0.022, p = 0.881, d.f. = 1; logistic regression analysis).

Because climbing on shrubs is related to scorpion size (the larger the scorpion, the lower the probability of climbing on a shrub) [24], and there was a negative correlation between scorpion size and size difference between the scorpion and its NELS, there was a potential bias in the results. To avoid this potential problem, we carried out the analysis for both a) the whole data set and b) data including only the size differences between scorpions of the two smaller size categories (size category 1: > 10–20 mm, and size category 2: > 20–30 mm, comprising more than 70% of all scorpions on shrubs) and their NELS, as there was no relationship between scorpion size and size difference in scorpions belonging to these size categories.

## Results

The density of scorpions/plot was higher in 2012 (17.46 ± 2.25 scorpions/plot) than in 2013 (9.07 ± 1.03 scorpions/plot) and 2014 (9.97 ± 1.13 scorpions/plot). However, the proportion of *B*. *occitanus* on shrubs was similar all three years (2012: 28.6%, 2013: 27.0%, 2014: 23.2%, χ^2^ = 3.07, p = 0.22, χ^2^ test). Most scorpions on shrubs were small individuals of size categories 1 (48.7% in 2012 to 65.2% in 2014) and 2 (20.3% in 2014 to 32.4% in 2013), these two size classes comprising 74.1–85.9% of the total number of individuals found on shrubs (Table 1). Scorpions on shrubs were observed using their usual sit-and-wait foraging strategy and 7.7% (25 out of 325) of the individuals on shrubs were found consuming prey. Identification of 22 out of these 25 prey as either epiphytic or ground dwelling [S1 Table] indicates that 10 of the 22 scorpions consuming prey on vegetation (45.5%) were feeding on prey most probably captured on the ground [S1 Table]. Thus, prey identified as either epiphytic or soil dwelling indicate that the proportion of prey captured on shrubs (24% of prey) and on the ground (76%) did not significantly differ from the proportions of scorpions observed on shrubs and on the ground (26.7% and 73.3%, respectively) (χ^2^ = 0.13, p = 0.72; χ^2^ test).

**Table 1:** Percentage of individuals in shrubs (%S) and on the ground (%G) in the different size categories in the three years.

Size category	2012		2013		2014	
	% S	% G	% S	% G	% S	% G
13 to < 20 mm	48.7	40.5	53.5	41.7	65.2	46.3
20 to < 30 mm	25.4	9.3	32.4	10.9	20.3	11.8
30 to < 40 mm	19.5	22.7	7.0	12.0	8.7	14.0
40 to < 50 mm	6.4	11.0	4.2	6.8	5.8	6.6
50 to < 60 mm	0	12.6	2.8	24.0	0	20.5
≥ 60 mm	0	3.9	0	4.7	0	0.9

### Prey size in shrubs vs. ground

Arthropods were significantly larger on the ground than in the shrub canopy, both in samples from sticky traps (ground: 4.28 ± 0.15 mm; shrubs: 2.12 ± 0.11 mm; Welch = 60.197, p < 0.0001, d.f. = 1, 243.83; Welch test on ranks) and on direct sampling by shrub beating and soil samples (ground: 4.16 ± 0.34 mm; shrubs: 2.54 ± 0.21 mm; Welch = 14.853, p = 0.0002, d.f. = 1, 171.44; Welch test on ranks). Similarly, the mean size of prey captured by small 13–30 mm scorpions on shrubs [6.26 ± 0.93 mm, ranging from 3 mm of *Clubiona* sp. spiders to 11.5 mm of a *Camptopus lateralis* (Germar, 1817) Hemiptera] was smaller than prey size on the ground [11.36 ± 1.63 mm, ranging from 3 mm of some spiders to 27 mm of an *Alphasida oberthueri* (Escalera, 1901) Tenebrionidae larvae] (Welch = 5.923, p = 0.023, d.f. = 1, 24.92; Welch test on ranks).

### Seasonal variations in shrub climbing

**Fig 1:**
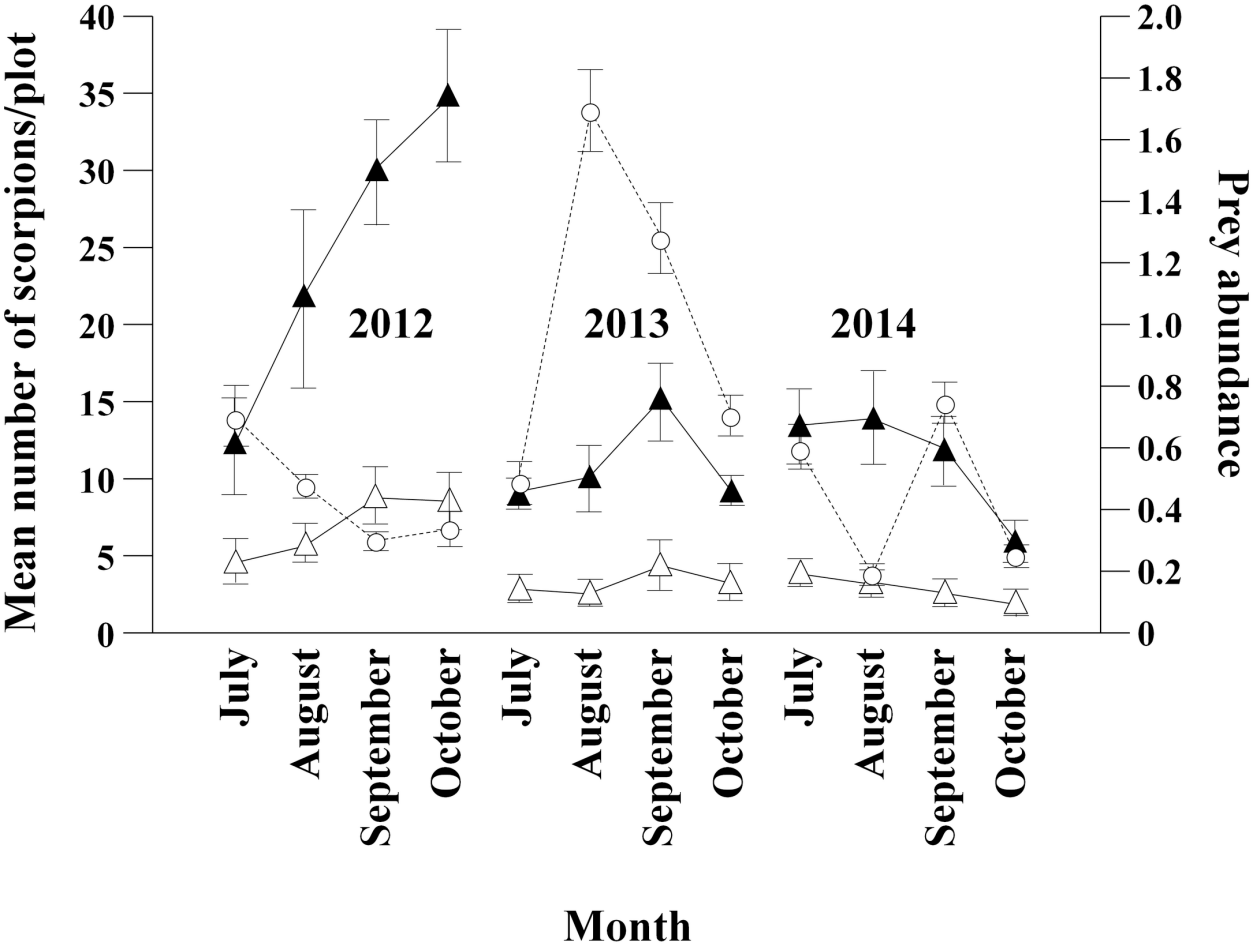
Seasonal variations of total number of active scorpions, number of scorpions on shrubs and prey availability in shrubs. Black triangles = Mean number of active scorpions/plot; White triangles = Mean number of scorpions on shrubs/plot; White circles = Mean number of prey/trap.

The proportion of *B*. *occitanus* on shrubs differed significantly among months in 2012 due to the higher frequency of scorpions found on shrubs during June (50% of the individuals) than in the other months (28.3%; χ^2^ = 10.47, p = 0.033, d.f. = 4). There were no seasonal differences in the proportion of scorpions on shrubs in the other two years (2013: χ^2^ = 1.96, p = 0.742, d.f. = 4; 2014: χ^2^ = 2.11, p = 0.716, d.f. = 4; Chi-square test) (Fig 1). In addition, there were no seasonal differences in the proportion of the different shrub species used by *B*. *occitanus* each year (2012: χ^2^ = 39.40, p = 0.50, d.f. = 40; 2013: χ^2^ = 32.92, p = 0.24, d.f. = 28; 2014: χ^2^ = 45.33, p = 0.26, d.f. = 40; Chi-square test) [S2 Table]; The same result was found pooling the data from the three years (χ^2^ = 39.92, p = 0.474, d.f. = 40).

The number of scorpions in shrubs was significantly related to the total number of active scorpions, but not to prey availability in shrubs (Full model: χ^2^ = 7.1534, p = 0.028, d.f. = 2; Effects: Number of prey: Estimate = -0.042 ± 0.241, χ^2^ = 0.0305, p = 0.8612; Total number of scorpions: Estimate = 0.721 ± 0.241, χ^2^ = 6.666, p = 0.0098, d.f. = 1; GLM) (Fig 1). Monthly variations in shrub selectivity and prey availability were not correlated, either (r_s_ = -0.147, p = 0.098, n = 128; Spearmann rank correlation).

### Shrub selectivity vs. prey availability

**Fig 2:**
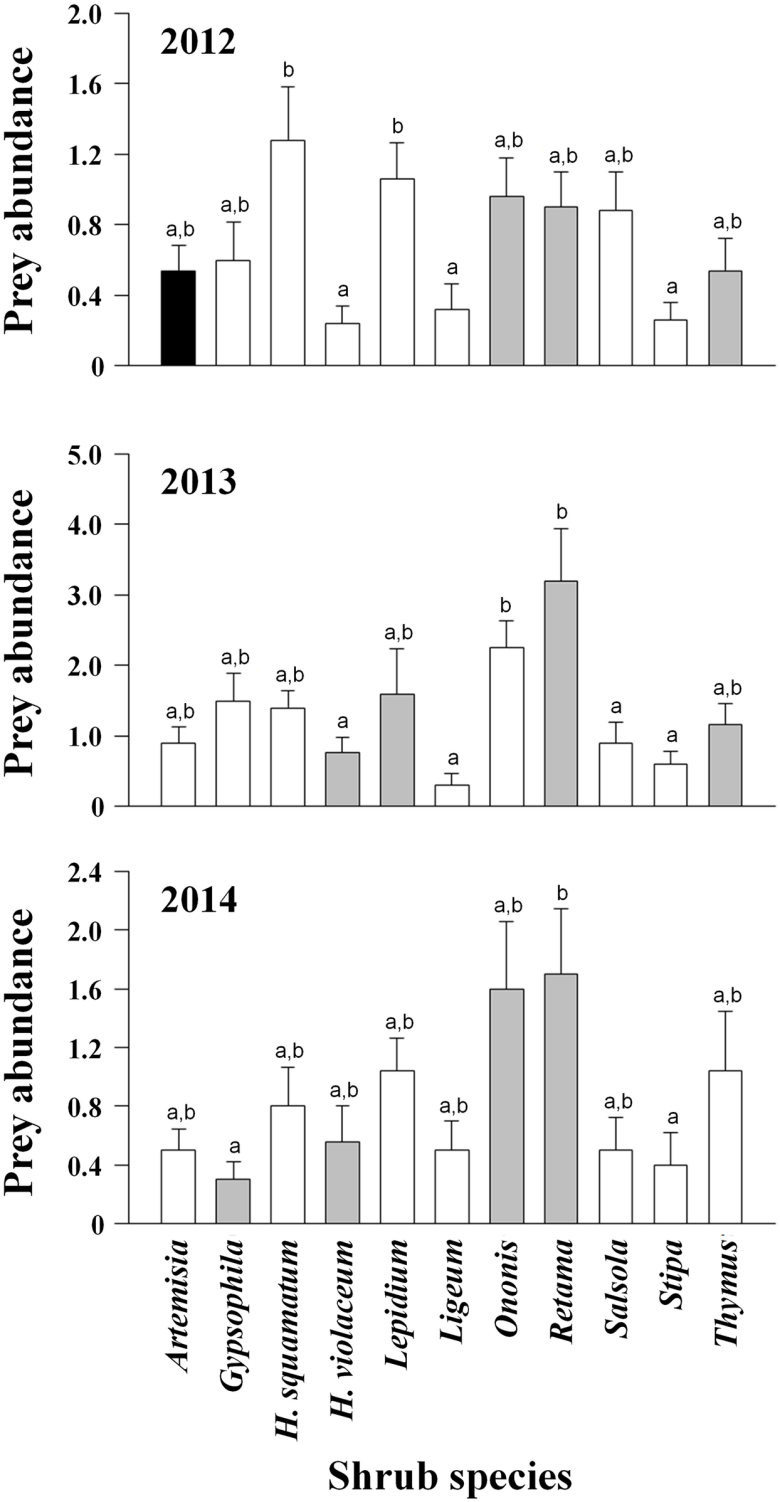
Prey abundance (arthropods/trap) on sticky traps in the different shrubs in 2012, 2013 and 2014. Gray, black, and white bars indicate shrubs positively selected, negatively selected and randomly used by *Buthus occitanus*, respectively. Different letters on top of each bar indicate statistically significant differences (REGWQ *post-hoc* test).

There were differences among years in the proportion of the different shrub species used by the scorpions (χ^2^ = 43.76, p = 0.0016, d.f. = 20; Chi-square test). Only in four shrub species was selection by scorpions significantly higher or lower each year (Table 2). As shown in Table 2 (see also Fig 2), only *Retama* was positively selected all three years, the selection of shrub species varying between years.

**Table 2:** Shrub selectivity (Savage’s index, *W*_*i*_) by *Buthus* cf. *occitanus* in 2012, 2013 and 2014.

Shrub	% used	% available	*W*_*i*_	S.E. (*W*_*i*_)	χ^2^	p
**2012**						
*Artemisia* spp.	12.2	29.6	0.41*	0.21	7.60	0.006
*Gypsophila struthium*	0.6	0.5	1.20	1.12	0.03	0.863
*Helianthemum squamatum*	12.8	21.2	0.60	0.21	3.47	0.063
*Helianthemum violaceum*	2.9	1.2	2.42	0.56	6.28	0.012
*Lepidium subulatum*	2.9	7.1	0.41	0.46	1.63	0.202
*Ligeum spartum*	4.7	8.6	0.55	0.37	1.53	0.216
*Ononis tridentata*	1.2	0.2	6.00**	1.11	20.23	< 0.0001
*Retama sphaerocarpa*	12.2	0.3	40.67**	0.74	2881.02	< 0.0001
*Salsola vermiculata*	11.6	7.1	1.63	0.25	6.26	0.0123
*Stipa tenacissima*	6.4	4.4	1.45	0.34	1.75	0.186
*Thymus zygis*	32.6	19.7	1.65*	0.13	23.55	< 0.0001
**2013**						
*Artemisia* spp.	11.1	20.1	0.55	0.23	3.79	0.052
*Gypsophila struthium*	0	0.6	0	-	-	-
*Helianthemum squamatum*	0	8.9	0	-	-	-
*Helianthemum violaceum*	11.1	4.4	2.50**	0.28	28.42	< 0.0001
*Lepidium subulatum*	11.1	5.5	2.02**	0.27	14.32	0.0002
*Ligeum spartum*	1.85	10.6	0.18	0.57	2.12	0.145
*Ononis tridentata*	1.85	0.7	2.51	0.72	4.43	0.035
*Retama sphaerocarpa*	3.7	0.4	8.80**	0.71	119.20	< 0.0001
*Salsola vermiculata*	0	6.7	0	-	-	-
*Stipa* spp.	7.41	6.0	1.23	0.31	0.56	0.454
*Thymus zygis*	51.85	36.1	1.44**	0.09	23.52	< 0.0001
**2014**						
*Artemisia* spp.	17.5	20.6	0.85	0.25	0.35	0.554
*Gypsophila struthium*	1.3	0.1	8.93**	1.42	31.33	< 0.0001
*Helianthemum squamatum*	7.5	7.0	1.07	0.42	0.03	0.956
*Helianthemum violaceum*	3.8	1.1	3.29**	0.67	11.79	0.0006
*Lepidium subulatum*	7.5	6.6	1.14	0.42	0.11	0.740
*Ligeum spartum*	5.0	14.6	0.34	0.50	1.75	0.186
*Ononis tridentata*	5.0	0.4	11.63**	0.75	198.76	< 0.0001
*Retama sphaerocarpa*	3.8	0.9	4.36**	0.70	23.26	< 0.0001
*Salsola vermiculata*	3.8	3.0	1.25	0.61	0.17	0.680
*Stipa tenacissima*	3.8	6.1	0.61	0.59	0.44	0.507
*Thymus zygis*	41.3	39.6	1.04	0.14	0.09	0.764

* Significant negative selection,

** Significant positive selection (χ^2^ test, P < 0.05 after sequential Bonferroni correction). Bold cases highlight significant (positive and negative) selection.

Prey abundance differed significantly among shrub species all three years (2012: Welch = 3.623, p = 0.0003, d.f. = 10, 119.64; 2013: Welch = 5.504, p < 0.0001, d.f. = 10, 84.88; 2014: Welch = 2.864, p = 0.004, d.f. = 10, 83.80; Welch test on ranks) (Fig 2). In 2012 and 2014 there was no relationship between prey availability and shrub selectivity by scorpions (2012: R^2^ = 0.008, F = 0.08, p = 0.790, d.f. = 10; 2014: R^2^ = 0.19, F = 2.14, p = 0.178, d.f. = 10; linear regression); Although, in 2013 the relationship was significant (R^2^ = 0.62, F = 14.58, p = 0.004, d.f. = 10; linear regression), this result was determined by the excentric selectivity value of *Retama sphaerocarpa*, and exclusion of this value from the analysis resulted in no significant relationship between prey availability and shrub selectivity (R^2^ = 0.13, F = 1.16, p = 0.313, d.f. = 9; linear regression). These results are corroborated by prey abundance in the different shrubs (Fig 2): shrubs with similarly low prey abundances were positively and negatively selected (e.g., *Artemisia* and *Thymus* in 2012); contrarily, shrubs differing significantly in prey abundance showed positive selectivity values (e.g., *Retama* vs. *Helianthemum violaceum* in 2013, *Retama* vs. *Gypsophila* in 2014).

### Relationship of size differences and distance on shrub climbing

**Fig 3:**
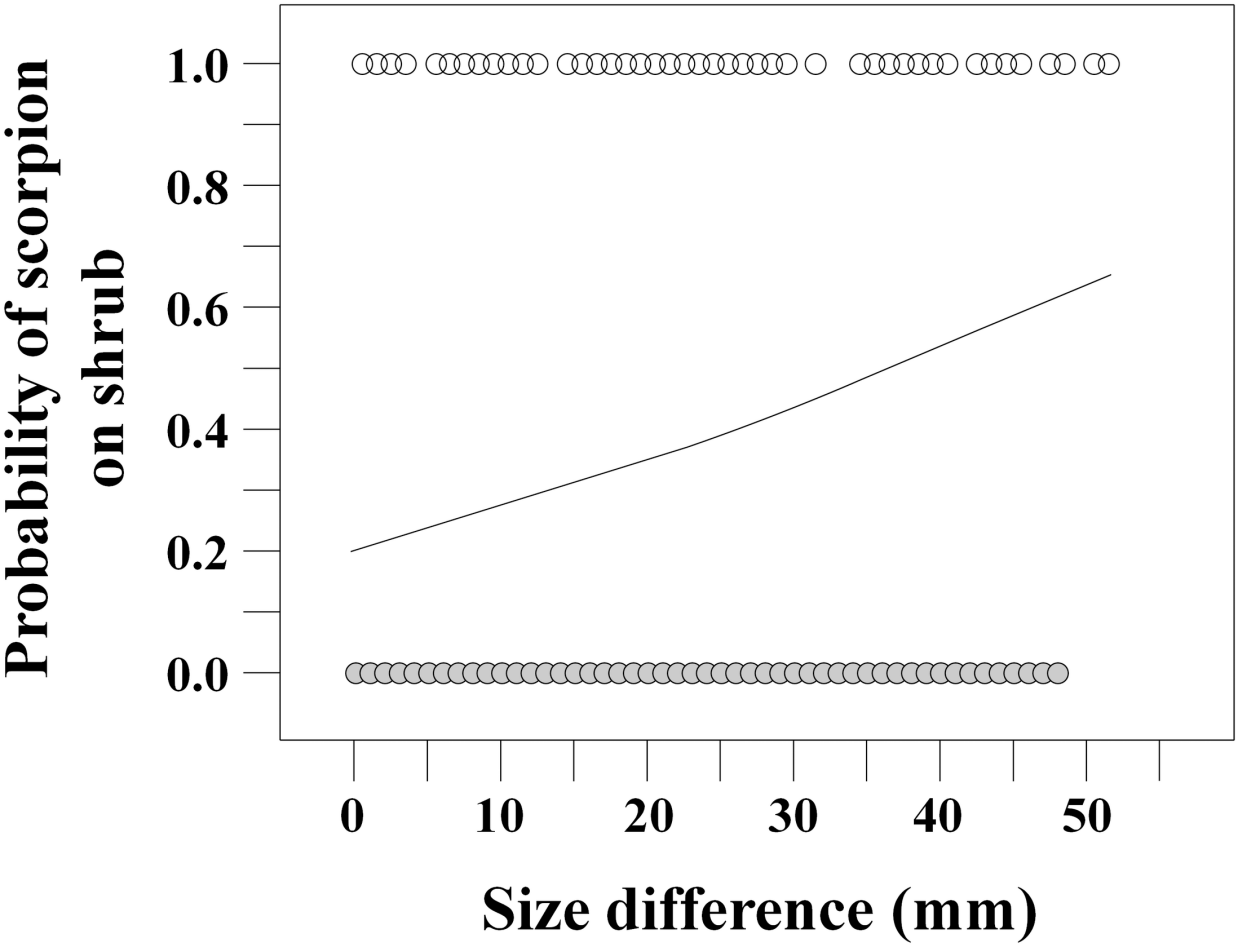
Scatterplot of *Buthus* cf. *occitanus* occurrence on shrubs (white circles) and on the ground (grey circles) in relation to size differences between a scorpion and its nearest neighbor of equal or larger size (NELS), and predicted probability curve of scorpion occurrence on shrubs from the logistic regression model (Intercept: estimate = -1.19 ± 0.17, Wald χ^2^ = 49.03, p < 0.0001, d.f. = 1; Size difference: estimate = 0.028 ± 0.007, Wald χ^2^ = 16.09, p < 0.0001, d.f. = 1).

Diet data showed that cannibalism was relatively frequent at the study site: 6.8% of the total number of prey (and 8.3% considering only identified prey) recorded during the three years of the study were conspecifics. The multiple logistic regression considering the relationship of a) the size difference between a scorpion and its NELS, and b) the distance between them, indicated that in both 2012 and 2014 the probability of a scorpion being found on a shrub was positively related to size differences between scorpions and that distance was not related to the probability of a scorpion being on a shrub (Table 3). The same results were found when the analyses were made considering only scorpions of the two smallest size classes (1 and 2) and their NELS, and for the whole data set, including all size categories (Table 3). In both years, the probability of finding a scorpion on a shrub increased by three-fold (from ca. 0.2 to ca. 0.6) when a larger closest neighbor occurred (odds ratios 3.60–3.97; Fig 3).

**Table 3:** Results of multiple logistic regressions for the relationship of size difference between a scorpion and its nearest neighbor of equal or larger size (NELS) and distance between the scorpions on the probability for a scorpion to be on a shrub. Results are provided for individuals of the two smallest size categories (1 and 2) and for scorpions of all size categories (1–6) for the two years (2012 and 2014).

**2012**			
**Size categories 1 and 2**			
Full model	χ^2^ = 7.58	p = 0.026	d.f. = 2
Parameter estimates			
Intercept	-1.027 ± 0.304	χ^2^ = 11.44	p = 0.0007
Size difference	0.026 ± 0.010	χ^2^ = 7.40	p = 0.007
Distance	-0.028 ± 0.054	χ^2^ = 0.27	p = 0.602
**All size categories (1–6)**			
Full model	χ^2^ = 9.75	p = 0.008	d.f. = 2
Parameter estimates			
Intercept	-1.196 ± 0.274	χ^2^ = 19.11	p < 0.0001
Size difference	0.027 ± 0.009	χ^2^ = 9.51	p = 0.002
Distance	0.006 ± 0.042	χ^2^ = 0.02	p = 0.883
**2014**			
**Size categories 1 and 2**			
Full model	χ^2^ = 7.660	p = 0.022	d.f. = 2
Parameter estimates			
Intercept	-0.982 ± 0.424	χ^2^ = 5.35	p = 0.021
Size difference	0.033 ± 0.013	χ^2^ = 6.78	p = 0.009
Distance	-0.076 ± 0.079	χ^2^ = 0.92	p = 0.338
**All size categories (1–6)**			
Full model	χ^2^ = 7.66	p = 0.022	d.f. = 2
Parameter estimates			
Intercept	-0.944 ± 0.408	χ^2^ = 5.34	p = 0.021
Size difference	0.032 ± 0.012	χ^2^ = 6.70	p = 0.010
Distance	-0.077 ± 0.077	χ^2^ = 1.00	p = 0.318

Distance between nearest neighbors was similar in 2012 and 2014 (4.05 ± 0.19 m in 2012 and 3.93 ± 0.20 m in 2014).

## Discussion

Our results do not support the idea that smaller-sized scorpions climb onto shrubs because more adequate-sized prey are available on the foliage than on the ground [24]. The larger size of prey captured by small scorpions on the ground (11.4 ± 1.6 mm) than on shrubs (6.3 ± 0.9 mm) could reflect that foraging on the ground was more profitable, since a similar rate of prey capture would provide more food on the ground than on a shrub. Thus, the hypothesis that small scorpions benefit from capturing smaller, more adequate prey on shrubs, suggested by Brown & O’Connell [23] and Sánchez-Piñero *et al*. [24], was not supported by our results.

No evidence supports the hypothesis that seasonal variations in prey availability in shrubs are related to shrub climbing. On one hand, the number of scorpions on shrubs varied seasonally with changes in the number of active scorpions, and in fact the proportion of scorpions on shrubs did not differ significantly over the main activity season of *B*. *occitanus* in the study area. Only in June 2012 was there a higher proportion of scorpions on shrubs (50%) than in the other months of the same year (28% on average), a result probably related to the observation of a higher number of wandering adult males in the study area at this time than in any other censuses carried out during this study. On the other hand, the number of scorpions on shrubs was unrelated to the variations in the number of prey in shrubs. These results together indicate that shrub climbing by scorpions did not correlate with changes in prey availability. No relationship between seasonal variations in climbing on vegetation and prey availability have been reported, either, for *Centruroides vittatus* [20], [23]. Nonetheless, a more precise analysis of the use of specific shrubs, such as preferred *Retama* and *Ononis*, would be necessary to address this question.

Shrub selectivity did not support the hypothesis that climbing was primarily related to prey availability, either. Most shrubs were used only randomly, and exclusively in the case of *Retama sphaerocarpa* in 2013 and 2014 did positive selection correspond to a shrub species with significantly higher prey availability, while other shrub species with positive selection, such as *Lepidium subulatum and Thymus zygis* in 2013, did not differ in prey availability from most other shrubs. In addition, *Helianthemum violaceum* and *Gypsophila struthium* appeared as significantly, positively selected shrubs in 2013 and 2014, respectively. These species were not among those having higher prey abundance values, suggesting that these shrubs could be selected for traits unrelated to prey availability, such as shrub architecture. These results, altogether, suggest that shrub selection by *B*. *occitanus* may result from a combination of shrub traits, such as architectural features and prey abundance. Thus, the high selectivity of *Retama sphaerocarpa* and *Ononis tridentata* by scorpions could be related not only to their high prey availability, but also to structural or microhabitat characteristics. McReynolds [20] indicated that climbing on different plants by *Centruroides vittatus* was probably associated with diverse uses, as some plants were used mostly as shelter (*Opuntia engelmannii*, *Echinocereus enneacanthus*) and others for foraging (*Acacia rigidula*) or, mostly, for predator avoidance (carrying prey onto the vegetation to feed).

By contrast, a positive relationship was found between size differences of neighboring scorpions and the probability of finding a scorpion on a shrub. This result supports the hypothesis that climbing on shrubs is related to the risk of cannibalism, since: a) the proportion of cannibalism in the diet of *B*. *occitanus* at the study site was similar to the frequency of conspecifics in the diet of other scorpion species [12], [14], [15], [16]; and b) larger scorpions eat smaller ones, and the greater the size difference between the two interacting individuals, the lower the possibilities of the smaller individual to escape the encounter [6]. Size differences have been indicated as being related to the risk of predation and cannibalism by smaller individuals in other animals, such as fish [50], salamanders [8] and isopods [7]. Thus, climbing on shrubs appears to allow smaller scorpions to reduce the risk of cannibalism while foraging. This could also account for the observations of individuals on shrubs feeding on prey captured on the ground found in this study and reported elsewhere for *B*. *occitanus* [22] and other scorpion species [13], [20]. Therefore, climbing on shrubs by *B*. *occitanus* agrees with the idea that cannibalism is of paramount importance to explain the ecology and behavior of scorpions [51].

However, the logistic model also indicated a probability of finding a scorpion on a shrub of ca. 20% when there were no size differences between two neighboring scorpions. Several factors may explain this probability. First, very hungry individuals may be able to cannibalize same-sized or even larger victims, as reported in wolf spiders [52]. Second, nocturnal ground predators other than *B*. *occitanus* conspecifics are known to prey on this scorpion at the study site, such as the Moorish gecko *Tarentola mauritanica* (Linnaeus, 1758) [53], [54] and the black widow *Latrodectus lilianae* Melic, 2000 [55], likely increasing predation risk and shrub climbing.

Third, we measured the risk of cannibalism in a clearly simplistic way, looking exclusively for the potential risk represented by the closest neighboring scorpion. Nonetheless, another neighbouring scorpion could also influence shrub climbing, and in some cases a larger scorpion was in fact nearby, although it was not the closest neighbor. Also, in some cases we may not have detected large doorkeeping females, since in some cases they quickly hid inside their burrows as we approached.

Fourth, chemical communication in scorpions [56], [57], [58], [59] suggests that individuals could gather information about their neighbors, allowing them to evaluate predation risks and display antipredatory responses, as shown in wolf spiders [60]. Since chemical cues of adult females may last more than one day on the ground [61], active individuals on a given night may still respond to these signals left by a neighboring adult female even if she was not active the same night.

Finally, dispersing juveniles may climb onto vegetation to reduce the risk of being cannibalized while moving within unknown territories (see [62] and references therein). As dispersing juveniles may gather information about conspecifics inhabiting an area from chemical or other cues (see above), the probability that these individuals climb on vegetation would increase as the risk becomes greater, as shown by our data. In this context, climbing onto shrubs may constitute a selective behavioral trait during juvenile dispersal in a heterogeneous landscape of both cannibalism risk and prey availability.

In contrast to our hypothesis, distance between two scorpions had no significant relationship with the probability of a scorpion being found on a shrub. This result agrees with the finding of Holderied et al. [63] that the noise produced by walking *B*. *occitanus* scorpions was negatively correlated or not at all with body size, suggesting that ground and air vibrations produced by scorpions are actually not a reliable cue to avoid approaching cannibals. Further, scorpions are sit-and-wait foragers that usually hunt only a few meters around their burrows, depending on age class (2–7 m in *Paruroctonus mesaensis*, a scorpion species similar in size to *B*. *occitanus*) [64]. Foraging distances from the burrow may constrain the effect of distance, since scorpions forage mostly not far from their burrows at quite regular distances. Data from Polis et al. [64] indicates that foraging individuals of the different age classes hunt at distances usually from 1 m (all age classes) to 5.5–7 m (in the youngest and oldest age classes, respectively) from the burrow [64]. In addition, chemical cues left by the scorpions (especially by adult females) last for days on the ground (see above) and thus may provide information to neighboring individuals not related to the distance to (or even presence of) a potential cannibalistic individual on a given night. These factors together may explain why distance was not a significant factor in our analysis.

In summary, this study provides for the first time empirical support to the hypothesis that shrub climbing in the scorpion *Buthus occitanus* is related to predator (cannibal) avoidance. In addition, we corroborate previous results reported for another scorpion species, *Centruroides vittatus*, that the hypotheses of selection of shrubs to forage related to prey availability, and occurrence of smaller, more suitable prey for small scorpions on vegetation were not supported [20], [23]. Experimental studies will be necessary to demonstrate whether climbing on shrubs is a selective response to avoid or minimize cannibalism in this scorpion species.

## Supporting Information

S1 Table Prey captured by *Buthus* cf. *occitanus* during the study. Whether taxa are ground/soil (Soil) or shrub-canopy (Epiphytic) dwellers is indicated. Heterocera, Cercopidae and *Buthus* cf. *occitanus* have been observed foraging both at ground level and in shrub canopies. The solitary bee was captured by the scorpion inside its burrow.
(PDF)

S2 Table Number of *Buthus* cf. *occitanus* scorpions in the different shrub species each month. Art = *Artemisia* spp., Gyp = *Gypsophila struthium*, H.sq = *Helianthemum squamatum*, H.vi = *Helianthemum violaceum*, Lep = *Lepidium subulatum*, Lig = *Ligeum spartum*, Ono = *Ononis tridentata*, Ret = *Retama sphaerocarpa*, Sal = *Salsola vermiculata*, Sti = *Stipa tenacissima*, Thy = *Thymus zygis*.
(PDF)
