# Supplementary material for: Watch Out for Your Neighbor: Climbing onto Shrubs Is Related to Risk of Cannibalism in the Scorpion Buthus cf. occitanus
Source: PLoS One. 2016 Sep 21;11(9):e0161747. doi: 10.1371/journal.pone.0161747 (PMC5031422; doi:10.1371/journal.pone.0161747)
Supplement: S1 Table — Whether taxa are ground/soil (Soil) or shrub-canopy (Epiphytic) dwellers is indicated. Heterocera, Cercopidae and Buthus cf. occitanus have been observed foraging both at ground level and in shrub canopies. The solitary bee was captured by the scorpion inside its burrow. (PDF) [file pone.0161747.s001.pdf]

S1 Table. Prey captured by *Buthus* cf. *occitanus* during the study. Whether taxa are ground/soil (Soil) or shrub-canopy (Epiphytic) dwellers is indicated. Heterocera, Cercopidae and *Buthus* cf. *occitanus* have been observed foraging both at ground level and in shrub canopies. The solitary bee was captured by the scorpion inside its burrow.

**Taxa**	**Prey Epiphytic/Soil**	**Number of prey**	**Size (mm)**	**Scorpions on**	
				**shrub**	**ground**
Araneae					
Castianeira badia	Epiphytic	1	9.5	1	-
Cebrennus sp.	Soil	1	6.0	-	1
Clubiona sp.	Epiphytic	1	3.0	1	-
Dyctina sp.	Epiphytic	1	3.0	1	-
Iberesia sp.	Soil	2	11.3	-	2
Linyphia sp.	Epiphytic	2	3.0	2	-
Oonopinus angustatus	Soil	1	3.0	1	-
Selamia sp.	Soil	1	10	1	-
Steatoda albomaculata	Soil	1	7.0	1	-
Thanatus sp.	Soil	1	3.0	-	1
Coleoptera					
Alphasida sp. larvae	Soil	4	28.8 ± 2.2	4	-
Asidini larvae	Soil	1	21.0	1	-
Cardiophorus sp. larvae	Soil	1	14.0	1	-
Cycloderes glabratus	Soil	1	9.5	1	-
Morica hybrida larvae	Soil	2	20.5 ± 2.5	2	-
Pimelia sp. larvae	Soil	2	21.5 ± 8.5	2	-
Embioptera					
Haploembia palaui	Soil	8	8.8 ± 0.7	4	4
Hemiptera					
Camptopus lateralis	Epiphytic	1	11.5	1	-
Cercopidae	Epiphytic/Soil	2	6.0	1	1
Cicadellidae	Epiphytic	1	7.0	1	-
Dyctiopharidae	Epiphytic	1	8.0	1	-
Emblethis parvus	Soil	1	3.5	-	1
Plinthisus sp.	Soil	1	4.0	-	1
Rhopalus parumpunctatus	Epiphytic	1	6.0	1	-
Hymenoptera					
Apoidea (solitary bee)	Soil	1	-	-	1
Camponotus foreli	Epiphytic	1	6.5	1	-
Crematogaster auberti	Epiphytic	1	3.6	1	-
Messor sexual female	Soil	2	12.0	-	2
Lepidoptera					
Aglossa pinguinalis larvae	Soil	1	22.0	-	1
Agrotis sp. larvae	Soil	1	36.0	-	1
Heterocera	Epiphytic/Soil	3	14.7 ± 3.7	-	3
Lepidoptera larvae	Soil	1	7.0	-	1
Raphidioptera					

Harraphidia laufferi larvae	Epiphytic	1	11.0	1	-
Scolopendromorpha					
Scolopendra cingulata	Soil	1	40.0	1	-
Scorpionida					
Buthus occitanus	Epiphytic/Soil	5	30.6 ± 5.0	2	3
Soliphuga					
Gluvia dorsalis	Soil	1	18.0	1	-
Zygentoma					
Lepismatidae	Soil	2	5.5 ± 1.5	1	1
Unidentified prey	-	14	-	10	4
