# Supplementary material for: Watch Out for Your Neighbor: Climbing onto Shrubs Is Related to Risk of Cannibalism in the Scorpion Buthus cf. occitanus
Source: PLoS One. 2016 Sep 21;11(9):e0161747. doi: 10.1371/journal.pone.0161747 (PMC5031422; doi:10.1371/journal.pone.0161747)
Supplement: S2 Table — Art = Artemisia spp., Gyp = Gypsophila struthium, H.sq = Helianthemum squamatum, H.vi = Helianthemum violaceum, Lep = Lepidium subulatum, Lig = Ligeum spartum, Ono = Ononis tridentata, Ret = Retama sphaerocarpa, Sal = Salsola vermiculata, Sti = Stipa tenacissima, Thy = Thymus zygis. (PDF) [file pone.0161747.s002.pdf]

S2 Table. Number of *Buthus* cf. *occitanus* scorpions in the different shrub species each month. Art = *Artemisia* spp., Gyp = *Gypsophila struthium*, H.sq = *Helianthemum squamatum*, H.vi = *Helianthemum violaceum*, Lep = *Lepidium subulatum*, Lig = *Ligeum spartum*, Ono = *Ononis tridentata*, Ret = *Retama sphaerocarpa*, Sal = *Salsola vermiculata*, Sti = *Stipa tenacissima*, Thy = *Thymus zygis*.

Month	Art	Gyp	H.sq	H.vi	Lep	Lig	Ono	Ret	Sal	Sti	Thy
2012											
June	1	0	3	0	0	0	0	3	0	0	3
July	4	0	4	2	1	2	1	6	8	2	18
August	7	0	11	1	1	1	1	5	6	4	22
September	5	1	3	0	3	2	0	4	4	2	7
October	4	0	1	2	0	3	0	3	2	3	6
2013											
June	0	0	0	0	0	0	0	0	0	1	0
July	0	0	0	1	1	0	0	1	0	1	6
August	0	0	0	2	2	0	0	1	0	0	7
September	3	0	0	2	0	0	0	0	0	1	11
October	3	0	0	1	3	1	1	0	0	1	4
2014											
June	2	0	0	1	0	0	0	1	0	0	2
July	1	0	1	0	3	2	0	0	0	3	10
August	5	1	3	0	0	0	2	0	1	0	6
September	2	0	1	1	2	2	0	0	1	0	5
October	2	0	0	1	1	0	0	1	1	0	3
